# Spatiotemporal Analysis of Fentanyl-Associated Overdose Deaths in Chicago, IL, USA

**DOI:** 10.1007/s11524-025-00986-9

**Published:** 2025-06-05

**Authors:** Hyojung Kang, Kaylee Janakos, Csaba Varga

**Affiliations:** 1 Department of Health and Kinesiology, University of Illinois Urbana-Champaign, Urbana, IL 61802 USA; 2 Health Care Engineering Systems Center, University of Illinois Urbana-Champaign, Urbana, IL 61802 USA; 3 Information Science, University of Illinois Urbana-Champaign, Urbana, IL 61802 USA; 4 Department of Pathobiology, University of Illinois Urbana-Champaign, Urbana, IL 61802 USA; 5 Carl R. Woese Institute for Genomic Biology, University of Illinois Urbana-Champaign, Urbana, IL 61802 USA

**Keywords:** Fentanyl, Overdose deaths, COVID-19, Spatiotemporal analysis, Chicago

## Abstract

Overdose deaths involving fentanyl represent a major public health crisis in the USA. This study investigates the spatiotemporal dynamics of fentanyl-involved deaths before, during, and after the COVID-19 pandemic and examines how sociodemographic factors influence these deaths across geographic regions. Using a retrospective ecological approach, we analyzed data on ZIP code-level fentanyl-related deaths in Cook County, IL, between 2018 and 2023, obtained from the Medical Examiner’s Office and linked with sociodemographic data from the American Community Survey. We first mapped area-level death rates to assess their distribution and then conducted global and local clustering analyses to identify spatial autocorrelations and the locations of high- or low-death-rate areas. A geographically weighted Poisson regression (GWPR) model evaluated the associations between area-level fentanyl-related death rates and the area-level proportion of young adults, males, and individuals with at least a college degree, disability rate, and poverty rate. Spatial analyses found stronger spatial autocorrelations during (2020–2021) and after (2022–2023) the pandemic. Initially, high death rates were concentrated in the downtown area of Chicago, and they expanded to the surrounding areas during and after the pandemic. The GWPR model revealed that an increase in the area-level proportions of poverty, disability, and young adult residents increased the fentanyl-related death rates in most of the areas. Our findings highlight the urgent need to address the evolving dynamics of fentanyl-related overdoses through tailored public health interventions that account for the unique socioeconomic determinants of different regions. Importantly, a comprehensive approach to addressing differences in overdose death rates and their risk factors will be crucial to mitigating this public health crisis.

## Introduction

Fentanyl is a highly potent synthetic opioid, estimated to be 50 to 100 times stronger than morphine [1]. Over the past decade, fatal and non-fatal overdoses involving fentanyl have become a major public health crisis in the USA. According to the United States Drug Enforcement Administration, 379 million doses of illicit fentanyl were seized in 2022 alone [2]. Between 2013 and 2022, the age-adjusted rate of drug overdose deaths involving synthetic opioids, including fentanyl, fentanyl analogs, and tramadol, increased significantly, from 1 death per 100,000 population to 22.7 deaths per 100,000 [3]. This alarming trend is largely attributed to the rapid growth of illicit production enabled by reduced supplier costs [4]. Manufacturers frequently mix fentanyl with other substances, such as cocaine, heroin, and methamphetamine; this has led to both intentional and unintentional use of fentanyl, further exacerbating the overdose crisis [5–7].

Cook County, IL, which encompasses the city of Chicago and its suburbs, is the second-largest county in the USA, with an estimated population of 5.087 million in 2023 [8]. The fentanyl crisis has severely impacted the county. In 2022, nearly 2000 opioid-related overdose deaths were reported, of which over 91% involved fentanyl [9]. Cook County’s opioid overdose death rate of 38.5 per 100,000 people significantly exceeded the national average, highlighting the severity of the crisis in the region.

Many studies have examined individual-level factors associated with opioid overdose and deaths, including those involving fentanyl. For example, individuals with a history of substance use disorder or prior overdose are at significantly higher risk of opioid overdose [10–12]. Frequent drug use and polysubstance use also increase the likelihood of fentanyl involvement in overdoses [13]. In addition, mental health conditions, such as depression and anxiety, have been strongly linked to increased vulnerability to fentanyl overdose [2, 12, 14]. Demographic disparities also play a crucial role, with young adults, non-Hispanic (NH) Black individuals, and males being disproportionately represented in opioid-related mortality statistics [15, 16]. Environmental and socioeconomic factors, such as living in urban areas or experiencing economic hardship, are associated with elevated rates of opioid overdose deaths [17]. However, much of this research has focused on opioid use in general, encompassing both prescription and illicit opioids, without specifically examining fentanyl.

Beyond individual-level factors, assessing the geographical variability of fentanyl overdose is critical for identifying areas that require prioritized resource allocations and designing targeted interventions tailored to the unique needs of high-risk regions. Previous studies have examined geographical patterns in opioid use and overdose deaths and evaluated their associations with social determinants of health. These studies indicate that factors such as area-level economic status, built environmental conditions, and the availability of healthcare resources for substance use disorders are linked to opioid-related mortality [18] [19] [20] [21] [22]. However, few studies have specifically investigated how these geographical and environmental factors are associated with overdose deaths exclusively involving fentanyl and its analogs.

Building on these identified gaps, this study aims to provide a more comprehensive understanding of the spatiotemporal dynamics of fentanyl-involved deaths in Cook County, IL. By analyzing patterns of fentanyl-related mortality across three periods—before, during, and after the COVID-19 pandemic—we address the need for specificity in understanding fentanyl and its analogs, distinct from broader opioid-related trends. To explore how these deaths are distributed across space and time, we conducted global and local cluster analyses, one for each of the three periods. Global analysis helps assess the extent of clustering of the high or low death rates across the region as a whole, while local analysis pinpoints specific areas with higher or lower-than-expected rates and offers more granular insight into area-level patterns [23]. Furthermore, we examine how sociodemographic factors influence deaths involving fentanyl. By illuminating these relationships, we aim to support urban health initiatives by informing targeted interventions and policies that address the unique needs of vulnerable populations in urban and other high-risk settings.

## Methods

### Data Source

We used publicly available data from the Cook County Medical Examiner’s Office to identify fentanyl-related deaths for the period spanning January 1, 2018, to December 31, 2023 [24]. This case archive provides detailed information on fatalities under the medical examiner’s jurisdiction or those reported to the office from Cook County, IL. For this study, we included cases for which fentanyl or its analogs (e.g., carfentanil, 4-ANPP, furanyl fentanyl, acetyl fentanyl, and acryl fentanyl) were identified as the primary cause of death. Also, we extracted information on the date and location of these incidents (i.e., where the death occurred) from the database and aggregated the data by ZIP code and year. A ZIP code is a five-digit numeric code the United States Postal Service uses to identify a specific postal delivery area.

To understand contextual factors related to fentanyl-related deaths, we used ZIP code–level population estimates and sociodemographic variables from the 5-year American Community Survey (ACS) covering 2018 to 2022 [25]. The variables included in this study were age distribution ((percentages of juveniles (5–17 years), young adults (18–39 years), adults (40–64 years)), gender (percentage of males), race/ethnicity (percentages of non-Hispanic Black and Hispanic populations), education (percentage of individuals with a college degree or higher), disability rate, poverty rate, and unemployment rate. For population estimates, we selected year-specific data corresponding to each study year from 2018 to 2022. For example, the 2018 ACS 5-year estimates (2014–2018) were used for the 2018 population counts. Since the 2023 ACS estimates (2019–2023) were not available at the time of analysis, the 2022 estimates were used as a proxy for that year. All variables were joined to the administrative boundary shapefile of Cook County ZIP codes obtained from the TIGER/Line database (www.census.gov).

### Data Analysis

All map creation and spatial analysis were conducted in ArcGIS Pro 10.7.1 (Environmental Systems Research Institute, Inc., Redlands, CA, USA). We employed a stepwise spatial analytical approach [26, 27] to detect areas within Cook County with high fentanyl-associated death rates and to identify their sociodemographic risk factors. We first built choropleth maps for 2-year mean incidence rates (IR) per 100,000 person-years for three periods: before the COVID-19 pandemic (2018–2019), during the COVID-19 pandemic (2020–2021), and after the COVID-19 pandemic (2022–2023). Then, we performed global and local clustering analyses for the same periods. Finally, we conducted a geographically weighted Poisson regression analysis for the entire period (2018–2023). These analyses were performed at the level of ZIP codes, a well-defined geographical unit commonly used for administrative and statistical purposes. The shapefile was projected to the NAD 1983 UTM Zone 16 N coordinate system for spatial analysis to ensure accurate geographic representation.

### Mapping Overdose Death Rates

We constructed choropleth maps for each of the three periods, using geometric intervals to categorize the IRs into five classes. The geometric interval categorization gives an approximately equal class width and a consistent frequency of observations per class. The categorization was slightly changed by the inclusion of a class representing an IR of 0. To make the IRs comparable over the three periods, the first period’s (2018–2019) class breakpoints were used for the two subsequent periods (2020–2021 and 2022–2023), keeping only the highest-class upper IR intact.

In addition, to account for the small-area problem, when the IRs in ZIP codes with small populations became unstable, we used the spatial empirical Bayes method with first-order queen contiguity weights to smooth these rates [28]. The queen contiguity defines neighbors that share a common edge and/or vertex and is recommended in areas, like Cook County, where ZIP code areas are irregularly shaped.

### Global Cluster Analysis

We used the Incremental Spatial Autocorrelation (Global Moran’s *I*) tool to evaluate the degree of spatial clustering of fentanyl-related death rates across progressively increasing distances [29]. This tool uses a fixed-distance band to conceptualize spatial relationships and calculate a Moran’s *I* value and a *z*-score with a *p*-value at each distance. This analysis begins with a default distance that ensures each feature (ZIP code area) has at least one neighbor. The distance band yielding the highest Moran’s *I* value was identified and subsequently used for the local cluster analysis. Moran’s I ranges from − 1 to + 1, where values near + 1 indicate strong positive spatial autocorrelation, meaning that similar values cluster together spatially. Conversely, values near − 1 indicate strong negative spatial autocorrelation, such that dissimilar values cluster in space. A value close to 0 suggests a random spatial pattern, with no evidence of clustering.

### Local Cluster Analysis

Then, we identified areas with high, low, or dissimilar fentanyl-related death rates by using the local Moran’s *I* method [30]. The zone of indifference parameter for the conceptualization of spatial relationships was used for the local cluster analysis. This conceptualization method gives a maximum weight for a specified distance band, and as this distance is passed, a gradually decreasing weight is given.

The local Moran’s *I* statistic identifies high-high IR areas (areas with high IRs are surrounded by areas with high IRs), low-low IR areas (areas with low IRs are surrounded by areas with low IRs), and outlier areas (high-low and low–high IR areas). A positive statistically significant (*p*-value ≤ 0.05) Moran’s *I* value signifies that the target area is surrounded by areas with identical IRs (high-high or low-low), and a significant negative local Moran’s *I* value indicates areas with dissimilar IRs (high-low or low–high).

### Geographically Weighted Poisson Regression

We conducted a geographically weighted Poisson regression (GWPR) analysis to examine sociodemographic factors associated with fentanyl-related overdose deaths at the ZIP code level. The dependent variable was the number of fentanyl-related deaths in each ZIP code area over 6 years (2018–2023), offset by the population size during the same period. Sociodemographic factors obtained from the ACS were initially considered potential independent variables, but a subset of the most significant variables was selected through exploratory regression.

This exploratory regression was performed using an ordinary least squares model, with selection criteria requiring a minimum adjusted *R*-squared of 0.5, a *p*-value ≤ 0.05 for all explanatory variable coefficients [31, 32], and a variance inflation factor not exceeding 7. Also, the model required acceptable residual normality as indicated by a Jarque–Bera test *p*-value of 0.1 and minimum spatial autocorrelation of residuals with a *p*-value of at least 0.1. Based on these criteria, we identified a subset of significant independent variables, which were incorporated into the GWPR analysis.

In the GWPR model, we used the golden search algorithm to determine the optimal bandwidth to determine the spatial scale over which the model weights nearby observations more heavily than those farther away. We did so by iteratively refining the search area based on the golden ratio, continuing until the lowest Akaike information criterion (AIC) value was reached. To account for local effects, a Gaussian weighting scheme was applied that assigns a maximum weight to focal features (e.g., ZIP code area) within the bandwidth, with the weight of neighboring features decreasing gradually as their distance from the focal features increases. For the GWPR model, parameter estimates for each explanatory variable and the intercept were computed and presented in maps to provide a visualization of their spatial distribution and enable assessment of their heterogeneity. The intercept coefficient represented the predicted value of the dependent variable (the fentanyl overdose rate) when all the independent variables (predictors) were set to zero.

## Results

### Descriptive Statistics

Table 1 provides an overview of the sociodemographic and economic characteristics of the 177 ZIP code areas included in the analysis. The average IR of fentanyl-related deaths between 2018 and 2023 was 26.01 per 100,000 persons (SD = 43.14). There were four ZIP code areas with 0 death events involving fentanyl. The population composition showed that adults (40–64 years) comprised the largest group, averaging 31.81% (SD = 4.35), followed closely by young adults (18–39 years) at 30.52% (SD = 10.97), indicating that working-age individuals represent most of the population in these ZIP codes. Males represented an average of 49.08% (SD = 3.08) of the population across ZIP codes. The percentage of the population identifying as non-Hispanic Black averaged 21.40% (SD = 28.48), while Hispanic individuals accounted for 21.05% (SD = 19.68). On average, 42.23% (SD = 23.09) of residents had a college degree or higher. Indicators of socioeconomic challenges included a disability rate of 10.72% (SD = 4.88), a poverty rate of 11.50% (SD = 7.83), and an unemployment rate of 9.11% (SD = 31.11).

**Table 1:** Summary statistics of sociodemographic variables for ZIP code areas, 2018–2023

Variable	Summary statistics
Number of ZIP code areas, *N*	177
Incidence rate per 100,000 persons between 2018 and 2023, mean (SD)	26.01 (43.14)
Percentage of juveniles (5–17 years), mean (SD)	15.76 (4.88)
Percentage of young adults (18–39 years), mean (SD)	30.52 (10.97)
Percentage of adults (40–64 years), mean (SD)	31.81 (4.35)
Percentage of older adults (65 + years), mean (SD)	16.39 (7.24)
Percentage of males, mean (SD)	49.08 (3.08)
Percentage of non-Hispanic black population, mean (SD)	21.40 (28.48)
Percentage of Hispanic population, mean (SD)	21.05 (19.68)
Percentage of individuals with a college degree or higher, mean (SD)	42.23 (23.09)
Disability rate, mean (SD)	10.72 (4.88)
Poverty rate, mean (SD)	11.50 (7.83)
Unemployment rate, mean (SD)	9.11 (31.11)

### Incidence Rate Maps

**Fig. 1:**
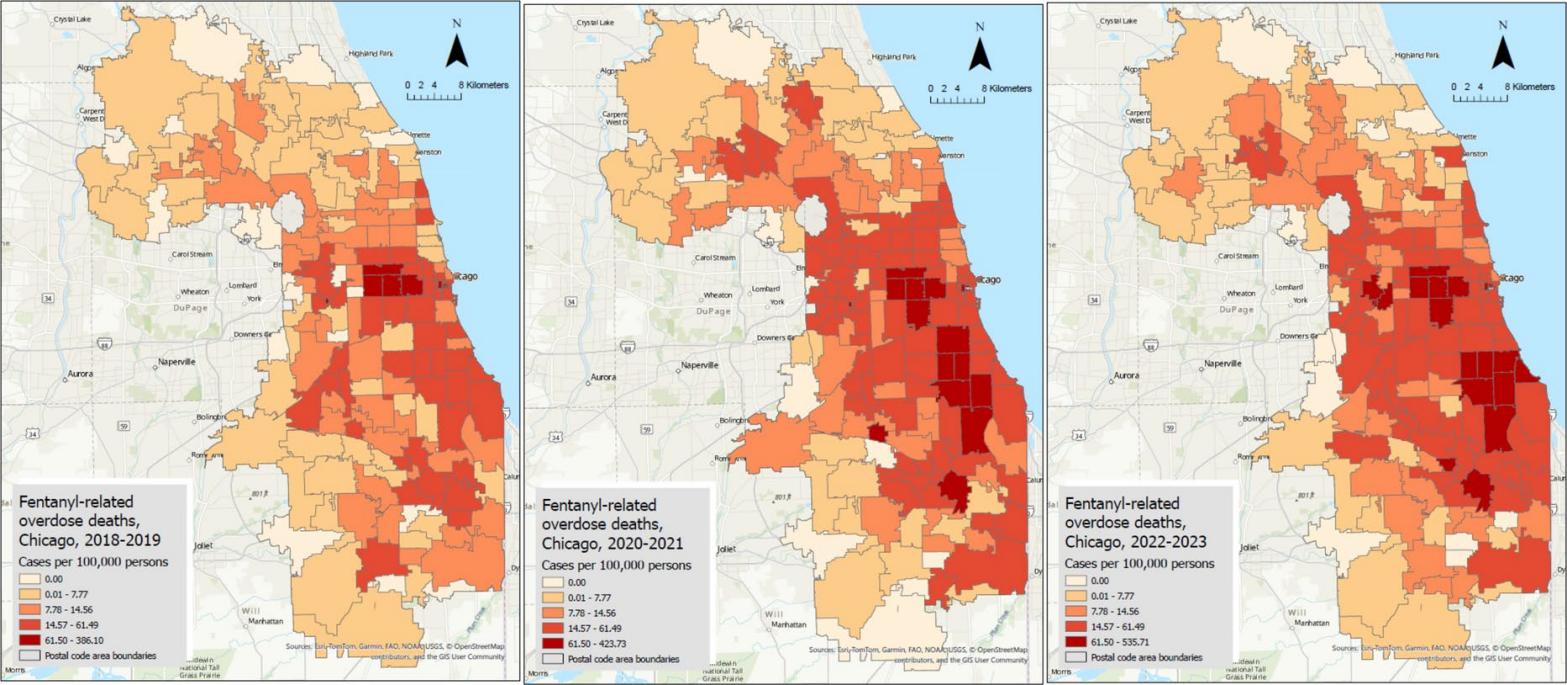
Maps for raw fentanyl-related death rates per 100,000 persons for three periods (2018–2019, 2020–2021, and 2022–2023)

Figure 1 shows the distributions of raw IR per 100,000 person-years across the three time periods. During 2018–2019, the IR ranged from 0 to 386.10 cases per 100,000 person-years, and the areas with the highest IRs were primarily concentrated in the western area of the city of Chicago, with moderate IRs observed across other regions of the city. During the COVID-19 pandemic (2020–2021), the IR ranged from 0 to 423.73 cases per 100,000 person-years, and a notable increase in IRs across the ZIP code areas was observed, with a pronounced rise in the southern parts of Chicago and surrounding regions. The western region, which already had high IRs in the earlier period, showed further increases. In the post-pandemic period, the IR ranged from 0 to 535.71 cases per 100,000 person-years, and the IRs continued to rise, but at a less dramatic rate than the transition from 2018 to 2019 to the COVID-19 pandemic period. The distribution patterns stabilized, with high IRs persisting in the western and southern areas. The northern regions and the suburbs exhibited relatively low IRs over the three study periods.

### Smoothed Incidence Rate Maps

**Fig. 2:**
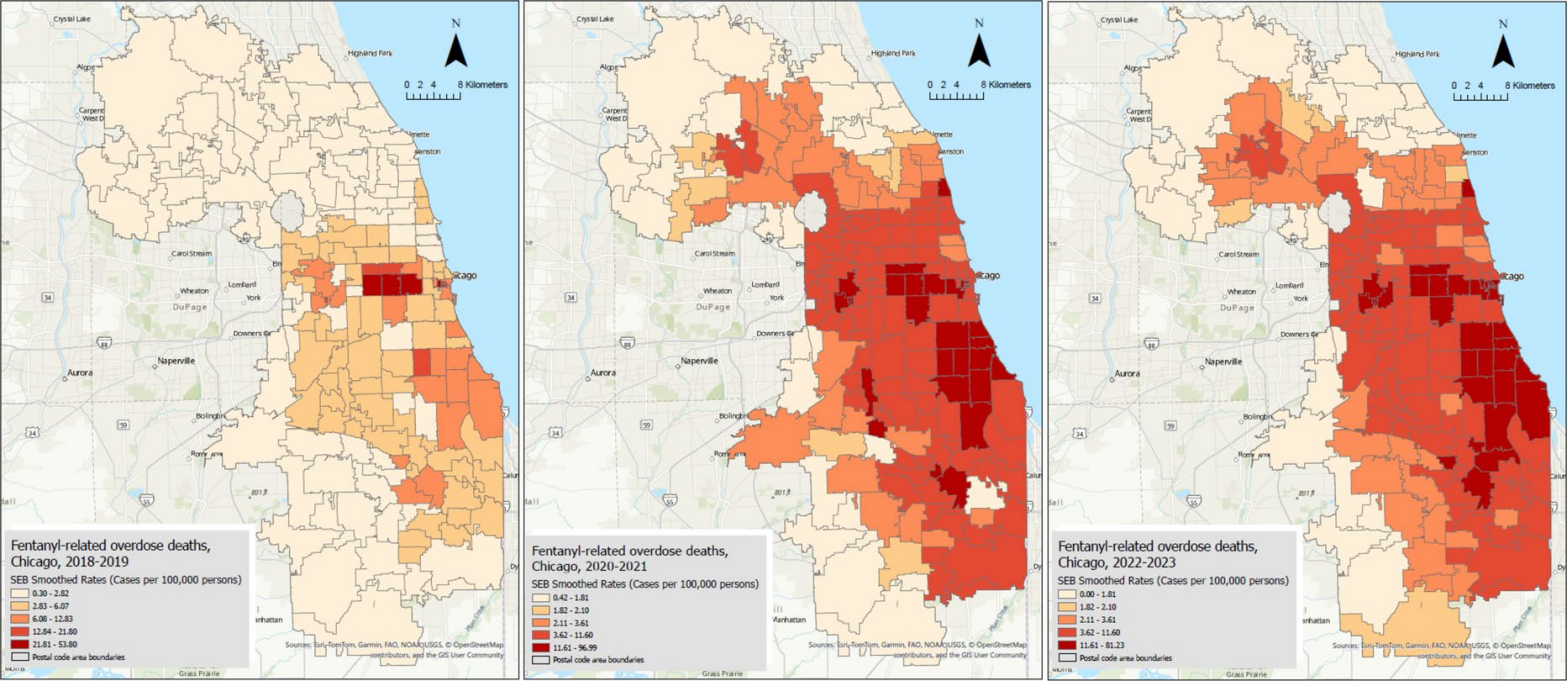
Maps for smoothed fentanyl-related death rates per 100,000 persons for three periods (2018–2019, 2020–2021, and 2022–2023)

The IRs of overdose deaths using the spatial empirical Bayesian (SEB) smoothing method are illustrated in Fig. 2. This smoothing technique borrows strength from neighboring areas’ IRs and the overall distribution of IRs, reducing extreme IRs, making the rates more reliable, and reducing the impact of outliers or regions with low sample sizes where the IRs tend to be overestimated. In the first study period (2018–2019), the highest IR was reduced to 53.8 cases per 100,000 person-years. Compared to the raw IR map, in the SEB smoothed map of the first period, the extent of high IR areas in the western area of the city of Chicago was reduced; however, three areas’ IRs remained high (28.91, 32.97, and 53.80), suggesting a stable high-rate area.

In the second period (2020–2021), the SEB smoothing reduced the highest IR to 96.99. Interestingly, this area, located in the western part of Chicago, was also identified in the first study period as the highest IR area. Additionally, in this period, the SEB smoothed maps followed the same patterns identified in the raw IR maps, keeping the extent of high-rate areas unchanged and showing an expansion of high-IR areas into the southern parts of the city of Chicago and surrounding regions. In the third period (2022–2023), the highest SEB smoothed IR was 81.23 in a ZIP code located in western Chicago, which was also identified as the highest IR area in the first and second periods. In addition, compared to the raw IR map of this period, the SEB smoothing increased the extent of high-rate areas, and in addition to the western part of Chicago, the high-rate areas included downtown Chicago and further areas in the southern region. The SEB smoothing method was useful to filter out noise (e.g., over- or under-estimated IRs) while preserving meaningful spatial structures (e.g., areas with high IRs).

### Global Clustering Analysis

For each period, we identified the distance band with the highest Moran’s *I* and *z*-score values. Across all three periods, the distance band was consistently 18,137 m, although the highest *z*-scores varied. The highest *z*-score was 6.53 in the first period, 9.22 in the second, and 8.34 in the third period. Details about the global clustering results are provided in Fig. 5 in the Appendix. All Moran’s *I* values were positive and were statistically significant (*p*-value < 0.05; *z*-score ≥  + 1.96) for all incremental distances, indicating significant spatial autocorrelation across all three time periods. The Moran’s *I* value was 0.110 in the first period, 0.240 in the second, and 0.112 in the third, indicating stronger spatial autocorrelation during the last two periods.

### Local Cluster Analysis

**Fig. 3:**
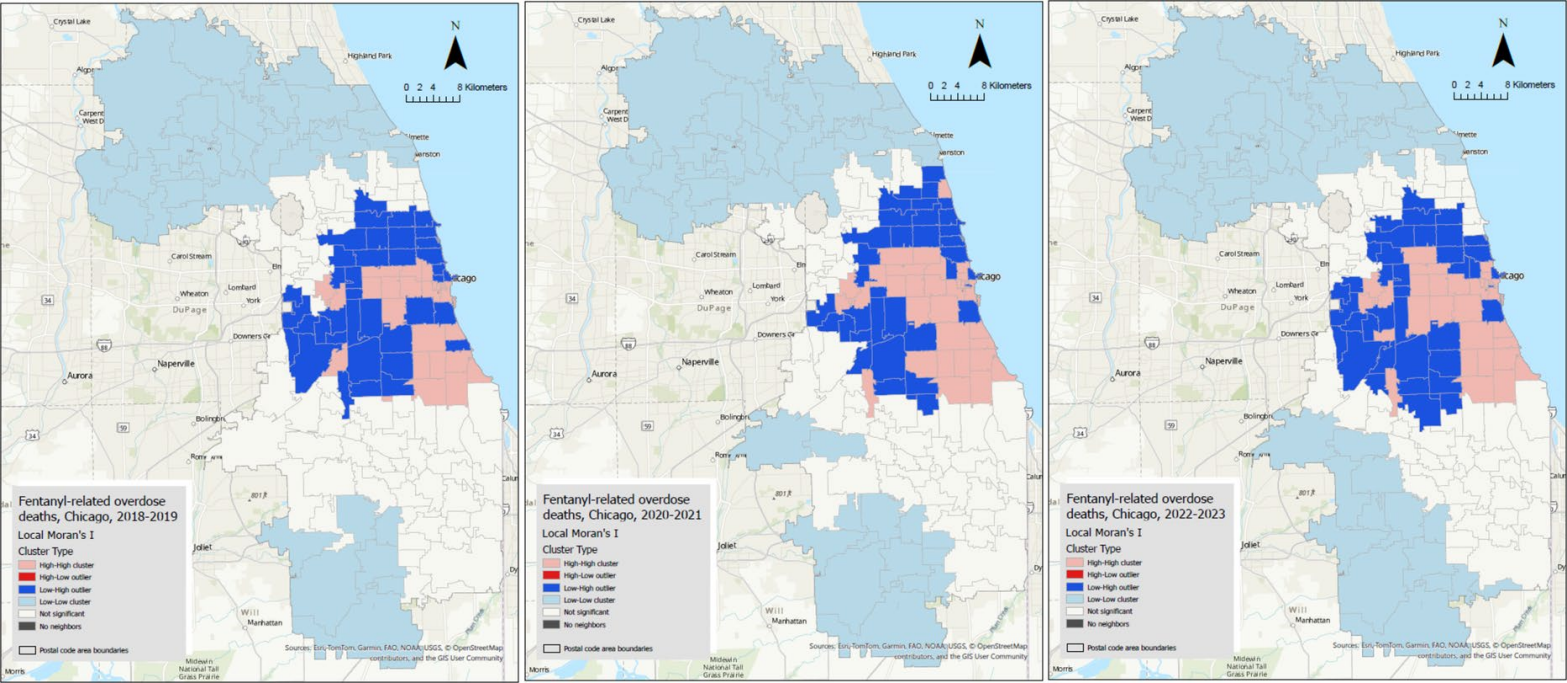
Maps illustrating the results of the local cluster analysis of fentanyl-related death rates for three periods (2018–2019, 2020–2021, and 2022–2023)

Local clustering analysis (Fig. 3) demonstrated the spatiotemporal dynamics of fentanyl-related deaths, highlighting the distribution of statistically significant hot spots (high-high clusters, shown in pink), cold spots (low-low clusters, shown in light blue), and outlier areas (high-low and low–high). Before COVID-19, hot spots were present but less extensive and were concentrated within the Chicago area. During the pandemic, a substantial expansion of these hot spots occurred, with a marked intensification and spread within the metropolitan area. Concurrently, the distribution of cold spots shifted. They were initially concentrated in the northern and southern fringes of Cook County (2018–2019), but the southern cold spots expanded significantly by 2020–2021, extending further northward toward the county’s central region. Figure 3 also suggests the presence of low–high outliers bordering the expanding hot spots, where future expansion of overdose deaths is expected. Interestingly, no high-low areas were identified, suggesting that the distribution of overdose deaths is consistent and increasing.

### Geographically Weighted Poisson Regression

**Fig. 4:**
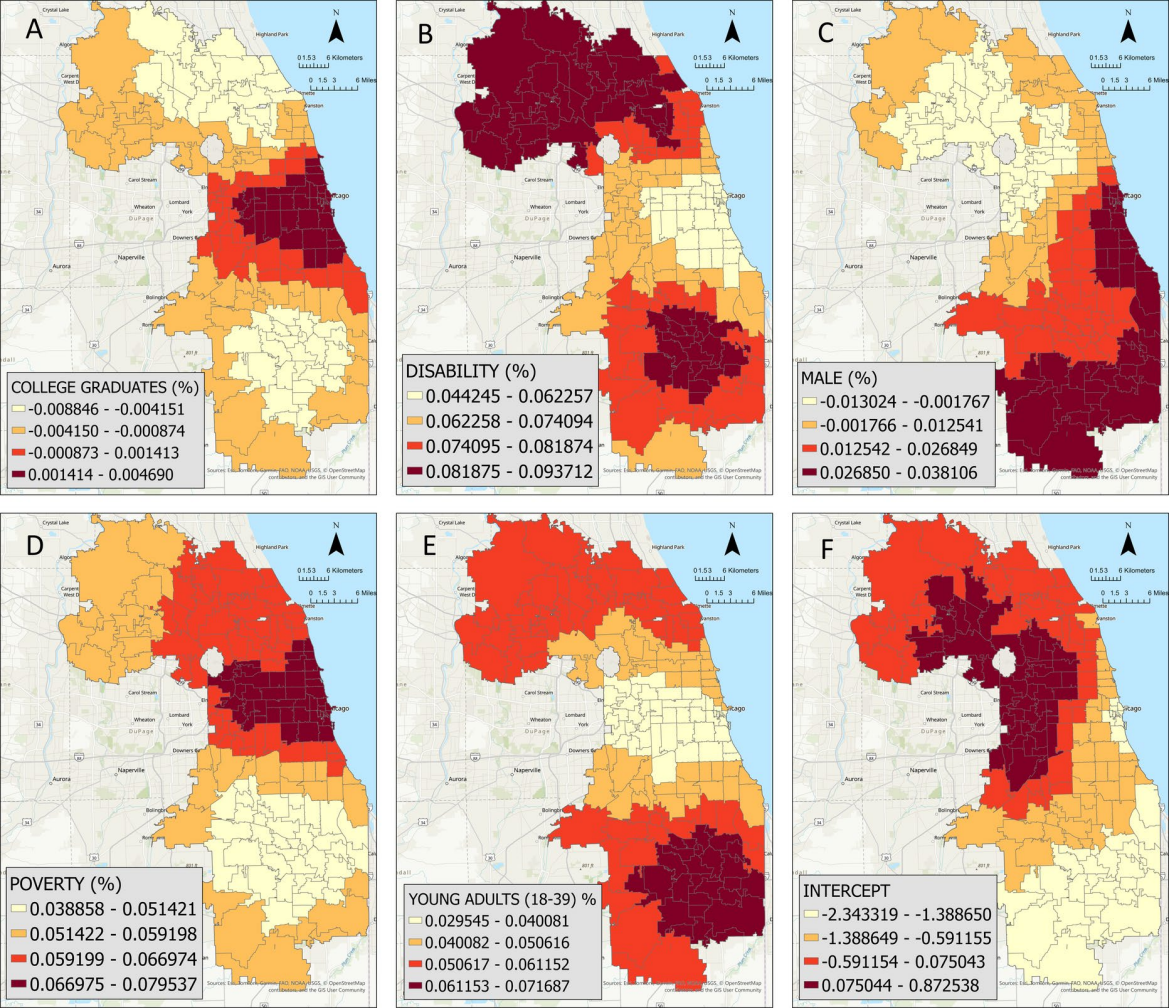
Coefficients of the explanatory variables

**Fig. 5:**
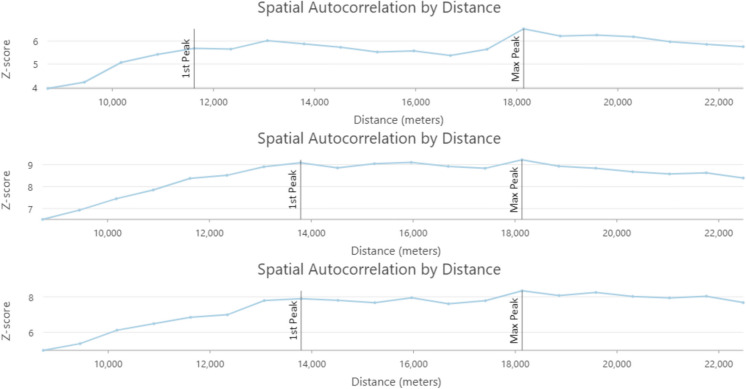
Spatial autocorrelation by distance bands: top (2018—2019), middle (2020—2021), bottom (2022—2023)

The best-fitting model (*R*^2^ = 0.62) identified from the exploratory regression analysis (a global model that does not account for the geographical variability in the outcome and predictor variables) included the following statistically significant explanatory variables (*p*-value < 0.001): percentage of young adults (18–39 years), percentage of males, percentage of individuals with a college degree or higher, disability rate, and poverty rate. Using these variables, the GWPR model (a local model that accounts for the geographical variability) explained 79% of the deviance in fentanyl-related mortality rates, showing a better fit than the global model. The variables initially considered but excluded from the GWPR model based on exploratory regression included the ZIP code level proportion of juveniles (5–17 years), the proportion of adults (40–64 years), the proportion of race/ethnicity (non-Hispanic Black and Hispanic populations), and the unemployment rate. The area-level poverty rate, disability rate, and proportion of young adults had a positive effect (i.e., with an increase in these predictors, the fentanyl-overdose death rate increases) in all areas (e.g., ZIP codes), while the area-level proportion of college graduates and the proportion of males had a positive effect in some areas and a negative effect (i.e., with an increase in these predictors, the fentanyl-overdose death rate decreases) in other areas. Figure 4 shows how the coefficients of the explanatory variables vary by ZIP code. For example, the proportion of males had a positive effect (red areas) in the southern part of Cook County, while it had a negative effect (blue areas) in the northern regions. The increase in the proportion of individuals with a college degree or higher had a positive effect on the death rate in the Chicagoland area (downtown Chicago and neighboring areas) and a negative effect in the southern and northern areas of Cook County.

The poverty rate had the highest positive influence on the fentanyl overdose death rates in the central and northern regions of Cook County, including the Chicagoland area, and its positive impact diminished in the southern regions of Cook County. The disability rate exhibited a stronger positive association with fentanyl overdose mortality rates in the northern and southern regions of Cook County, while the positive effect was weaker in the central region, particularly the Chicagoland area. The proportion of young adults had the highest positive effect on the fentanyl overdose death rate in the southern part of Cook County; the positive effect was reduced in the Chicagoland area. The distribution of the intercept coefficients (the predicted values of the fentanyl-overdose death rates when the predictor variables are not included in the model) had positive values in northern and central Cook County and negative values in the southern areas.

## Discussion

Our study used a stepwise spatial epidemiological approach to evaluate the area-level distribution and risk factors of fentanyl-related deaths. The highest mortality rates were identified in Chicagoland in the first period (2018–2019); then the high-rate areas extended southward and northward during the next two periods (2020–2021 and 2022–2023). The global cluster analysis indicated that fentanyl-related overdose deaths were not randomly distributed across Cook County but instead tended to cluster within a geographic range of approximately 18 km during all three periods. However, the larger Moran’s *I* values observed in the last two periods suggest that similar values (e.g., high overdose rates) became more strongly clustered geographically over time. The local cluster analysis identified statistically significant high-rate (hot-spot) areas in the Chicagoland area in the first period; those hot-spot areas expanded northward and southward during the next two periods. Cold-spot (low-rate) areas in the north of Cook County remained stable in all periods, while the cold-spot areas in the south expanded northward in the last two periods. By assessing the area-level distribution of the coefficients for the predictor variables of the GWPR model, we found that areas with a high proportion of poverty, disability, and young adults had higher fentanyl-related death rates in all areas of Cook County, while larger proportions of college graduates and males were associated with rising death rates in certain areas and decreasing death rates in other areas over time.

Previous studies have investigated trends of fentanyl-involved deaths in Cook County. For example, Denton et al. (2008) reported approximately 350 deaths between 2005 and 2007 [33] and Serinelli et al. (2019) documented 1244 deaths between 2015 and 2017 [34], reflecting a more than threefold increase in mortality over a decade. Building on that body of work, our study provides updated statistics using more recent data. Specifically, we identified 1788 deaths in 2018–2019, 3125 deaths in 2020–2021, and 3344 deaths in 2022–2023. These figures demonstrate a continued increase in fentanyl-involved overdose deaths and underscore the need for more effective and timely public health mitigation strategies.

The increasing trend of fentanyl-associated mortality in Cook County during the COVID-19 pandemic was noticeable, mirroring the nationwide pattern in the U.S. Between 2019 and 2020, the age-adjusted rate of drug overdose deaths involving synthetic opioids other than methadone nearly doubled, rising from 11.4 to 21.8 deaths per 100,000 population [35, 36]. Previous studies also documented similar rising trends in fentanyl-related overdose deaths during this period [37–40]. The significant increase in drug overdose deaths, primarily driven by fentanyl, during the pandemic can be attributed to various factors. The closure or limited availability of health care resources and treatment services left individuals who had substance use disorders without timely and essential support [41]. Also, limited access to prescription opioids, combined with the increased availability of fentanyl in the illicit drug supply, likely pushed some individuals to seek unregulated alternatives to manage pain [42]. Social-distancing measures and economic hardships further exacerbated mental health issues, leading to the initiation or escalation of substance use behaviors [43]. These findings underscore the need for multifaceted approaches to addressing the ongoing fentanyl crisis, including controlling the drug supply and improving access to health services and harm reduction programs. Expanded services (e.g., telemedicine and mobile health units) can ensure the continuity of treatment services during emergencies, while increasing the availability of harm reduction tools (e.g., naloxone and fentanyl test strips) can mitigate overdose risks.

The results from our mapping and clustering analysis revealed spatial heterogeneity in fentanyl-related overdose deaths in Cook County. During 2018–2019, 28 ZIP code areas, predominantly concentrated within the City of Chicago, were identified as hot spots. Notably, 23 of these hot spots persisted over the subsequent four years. This finding underscores the importance of proactive strategies to address the overdose epidemic in these locations. Once an overdose epidemic becomes entrenched, reversing its course is difficult. Also, we identified nine ZIP code areas that had low–high death rates (i.e., areas with low mortality surrounded by neighbors with high mortality) during 2018–2019 and transitioned to become hot spots during the COVID-19 pandemic, suggesting an expansion of the high-death-rate areas. Through such local analyses, public health officials can better identify at-risk areas and prioritize them for focused monitoring and preventive interventions. For example, they can strategically allocate resources such as naloxone distribution sites, harm reduction services, and mobile outreach units to both persistent and emerging hot spots. Our findings also highlight the importance of considering spatial relationships when addressing drug overdose as a public health issue. The spread of drug supply networks and dealers is not confined to specific regions but often extends into neighboring communities, which amplifies the chain effects of the epidemic. Incorporating spatial analytics into routine surveillance systems can help disrupt these patterns by enabling early detection of geographic shifts in overdose patterns and informing timely location-based responses.

Our findings from the GWPR model highlighted spatial heterogeneity in the relationships between sociodemographic factors and fentanyl-related overdose death rates. For example, the relationship between the percentage of residents with higher education and overdose death rates was positive in the City of Chicago and its surrounding areas, whereas it was negative in other areas of Cook County. Typically, higher levels of education act as a protective factor against overdose deaths, as populations with higher education are more likely to have stable employment and income, which reduces their exposure to socioeconomic conditions that contribute to illicit substance use or overdose [44, 45]. However, the divergent patterns observed in our study suggest that the urban context in the Chicago metropolitan area modifies this relationship. In urban areas, the protective effect of higher education at the community level may be weakened because of greater drug market penetration and increased access to fentanyl-laced substances. These findings point to the need for location-based substance use education and harm reduction strategies, even in relatively well-educated urban communities. For example, integrating drug-checking services into public health outreach and expanding educational efforts that inform communities about the risks associated with dynamically changing illicit drug markets (e.g., the emergence of new synthetic substances) may help address the unique overdose vulnerabilities among populations with higher socioeconomic indicators but elevated exposure to active drug supply networks.

The poverty rate, on the other hand, remained a consistent and significant risk factor for fentanyl-related overdose deaths. Poverty is a proxy for structural disadvantages, such as limited access to healthcare, poor housing conditions, and exposure to chronic stressors, all of which contribute to substance use vulnerability [17, 46–48]. Our study found that the relationship between poverty and overdose deaths was strongest in the Chicagoland area. This can be attributed to structural inequities, as the City of Chicago is a segregated city, with poverty concentrated in specific areas that affect minority communities [49]. In particular, Black and Hispanic neighborhoods face systemic disadvantages driven by concentrated poverty, such as underfunded schools and limited access to healthcare [50, 51]. These structural inequities exacerbate health risks and create a cycle of economic instability and substance use disorders. These findings emphasize the need for multi-sectoral urban health interventions that go beyond individual-level risk reduction. Policies that prioritize economic development in neighborhoods, such as affordable housing initiatives, job training programs, Medicaid expansion for behavioral health services, and expanded access to healthcare and harm reduction programs, can help reverse overdose trends in these affected communities. Several study limitations must be acknowledged. First, fentanyl-related deaths in Cook County may not be fully captured by the Medical Examiner’s Office data. While these data have been used in previous studies and provide an approximation of overdose death trends for the region, some cases may be underreported because of incomplete data, misclassification, or delays in reporting. Second, the study was conducted at the ZIP code level, which, while useful for identifying localized patterns, limits the granularity of the findings. Aggregating data at this level may obscure variations within communities that could contribute to fentanyl-related overdose death rates. Also, reliance on aggregated data carries the risk of ecological fallacy, wherein conclusions about individuals cannot be inferred from group-level findings. Finally, this study did not account for potential determinants other than sociodemographic factors, such as access to healthcare, law enforcement practices, and the availability of harm reduction services. These factors should be explored in future research.

In conclusion, this study found continuous, substantial increases in fentanyl-involved overdose deaths in Cook County, IL, between 2018 and 2023, with sharp increases during the COVID-19 pandemic period (2020–2021). These findings highlight the urgency of addressing the evolving dynamics of fentanyl-related overdoses during times of heightened societal stress. Moreover, this study demonstrates the utility of spatiotemporal analysis in identifying geographic variations in overdose mortality and pinpointing areas where prevention and harm reduction resources should be prioritized. To effectively combat the fentanyl overdose crisis, it will be crucial to leverage these insights to inform region-specific public health interventions that account for the unique sociodemographic and environmental factors contributing to overdose risks.

## Data Availability

All data supporting the findings of this study are available within the paper and its Supplementary Information.

## Appendix 2. Summary statistics of geographically weighted Poisson regression coefficients

**Table:**

	Mean (95% confidence interval)	Minimum	Maximum
College graduates (%)	− 0.0017 [− 0.0022, − 0.0012]	− 0.0088	0.0047
Disability (%)	0.073 [0.0711, 0.075]	0.0442	0.0937
Males (%)	0.0602 [0.0587, 0.0617]	0.0389	0.0795
Poverty (%)	0.0126 [0.0103, 0.0148]	− 0.013	0.0381
Young adults (%)	0.0486 [0.0471, 0.0501]	0.0295	0.0717
