# Correction: Spatiotemporal Analysis of Fentanyl-Associated Overdose Deaths in Chicago, IL, USA

**DOI:** 10.1007/s11524-025-00993-w

**Published:** 2025-06-30

**Authors:** Hyojung Kang, Kaylee Janakos, Csaba Varga

**Affiliations:** 1 Department of Health and Kinesiology, University of Illinois Urbana-Champaign, Urbana, IL 61802 USA; 2 Health Care Engineering Systems Center, University of Illinois Urbana-Champaign, Urbana, IL 61802 USA; 3 Information Science, University of Illinois Urbana-Champaign, Urbana, IL 61802 USA; 4 Department of Pathobiology, University of Illinois Urbana-Champaign, Urbana, IL 61802 USA; 5 Carl R. Woese Institute for Genomic Biology, University of Illinois Urbana-Champaign, Urbana, IL 61802 USA

**Correction: Journal of Urban Health**

10.1007/s11524-025-00986-9

The surname of coauthor Kaylee Janakos was misspelled in the article as originally published.

The original article has been corrected.

